# Negative Expression of DSG1 and DSG2, as Prognostic Biomarkers, Impacts on the Overall Survival in Patients with Extrahepatic Cholangiocarcinoma

**DOI:** 10.1155/2020/9831646

**Published:** 2020-08-10

**Authors:** Shu Xu, Shengfu Huang, Daiqiang Li, Qiong Zou, Yuan Yuan, Zhulin Yang

**Affiliations:** ^1^Department of General Surgery, The Second Xiangya Hospital, Central South University, Changsha, Hunan 410011, China; ^2^Department of Pathology, The Second Xiangya Hospital, Central South University, Changsha, Hunan 410011, China; ^3^Department of Pathology, The Third Xiangya Hospital, Central South University, Changsha, Hunan 410013, China

## Abstract

**Aims:**

To evaluate the expression of DSG1 and DSG2 and investigate their clinicopathological significance in EHCC.

**Method:**

The protein expression of DSG1 and DSG2 was measured by EnVision immunohistochemistry in 15 normal biliary tract tissues, 10 biliary tract adenoma tissues, 30 peritumoral tissues, and 100 EHCC tumour tissues.

**Result:**

The expression of the DSG1 and DSG2 proteins was significantly lower in EHCC tumour tissues than in normal biliary tract tissues, biliary tract adenoma, and peritumoral tissues (*P* < 0.05). Adenoma and peritumoral tissues with negative DSG1 and/or DSG2 protein expression exhibited atypical hyperplasia. DSG1 expression was positively correlated with DSG2 expression in EHCC (*P* < 0.01). In patients with good differentiation, no invasion, no lymph metastasis, TNM I + II stage, and radical surgery, the positive expression of DSG1 and DSG2 proteins was higher (*P* < 0.05). In comparison to patients with negative DSG1 and/or DSG2 expression, the average overall survival time of those with positive expression was significantly longer (*P* = 0.000). Cox multivariate analysis revealed that negative DSG1 and DSG2 expressions were independent of poor prognosis factors in EHCC patients. The AUC calculated for DSG1 was 0.681 (95% confidence interval: 0.594–0.768) and that for DSG2 was 0.645 (95% confidence interval: 0.555–0.734), while that for DSG1 and DSG2 was 0.772 (95% confidence interval: 0.609-0.936).

**Conclusions:**

Negative protein expression of DSG1 and DSG2 is closely related to the pathogenesis, severe clinicopathological characteristics, aggressive biological behaviours, and dismal prognosis in EHCC.

## 1. Introduction

Cholangiocarcinoma (CCA) occurs at any point of the biliary tree, deriving from epithelial cells of biliary ducts [1]. CCA is one of the most lethal solid tumours with poor prognosis, with only a 2% 5-year survival rate when it has spread outside the liver. It is a rare cancer comprising less than 2% of all cancers. CCA is classified as intrahepatic (IHCC) and extrahepatic CCA (EHCC). EHCC is the most common CCA and accounts for approximately 90% of CCA cases [1, 2]. The diagnosis of EHCC is based on clinical symptoms, blood testing, imaging, and histology and cytology [3]. In additional to traditional imaging, the main diagnostic method for EHCC includes endoscopic retrograde cholangiopancreatography (ERCP) [4]. To date, besides systemic chemotherapy and targeted radiation, surgical resection or liver transplantation is the only curative treatment for EHCC. Unfortunately, once progressing, recurring, or relapsing, the prognosis is very poor. This dismal prognosis is the result of both the inability to reliably detect EHCC at an early stage and the lack of successful therapies other than radical surgical excision. It is imperative to reveal valuable protein biomarkers that are closely related to the pathological features of EHCC.

Desmogleins (DSGs), as desmosome components, are critical for the structure of intercellular junctions and maintenance of epithelial barrier integrity. They may play a very important role in tumorigenesis and dysfunction of cell adhesion molecules in general. DSGs are also key regulators of signalling pathways involved in differentiation, epidermal homeostasis, and carcinogenesis. The loss of desmosome proteins promotes the process of epithelial-mesenchymal transition (EMT), linked to the formation of metastasis [5]. Desmosomes consist of a range of proteins from the plakin family, the armadillo family, and the cadherin family (DSGs and desmocollins). Many of these proteins have been found to be related to tumour emergence and progression. However, their role seems to be ambivalent. Some studies give evidence for a tumour suppressive role for the desmosome proteins, such as in lung cancer [5], oral intraepithelial neoplasms and cancer [6–8], head and neck cancer [9], prostate cancer [10], and gastric cancer [11], and the expression of DSG1 and DSG2 is low, which correlates with longer patient survival and/or inhibited tumour progression. Ramani et al. found that the levels of immunoreactivity of DSG1 and DSG2 were reduced in pancreatic adenocarcinomas compared with levels in both chronic pancreatitis tissues and normal pancreas. A reduction in the amount of the cell adhesion components DSG1 and DSG2 in pancreatic tumours suggests that loss of these desmosome proteins may play a role in pancreatic cancer invasion [12]. As reviewed by Dusek and Attardi, in other epithelial cancers (e.g., skin, head, and neck; gastric; colorectal; bladder; breast; prostate; and cervical cancers), desmosome proteins were downregulated or even lost and associated with dismal prognosis [13], while others suggest DSGs possess oncogenic function. For example, membrane negativity for DSG1 and cytoplasmic negativity for DSG1 are favourable markers for 5-year cancer-specific survival in SCCs of the anal region [14]. DSG2 is upregulated in malignant skin carcinomas [15] and human colon cancers [16]. The upregulation of DSG1 and/or DSG2 is related to enhanced tumour progression and/or shorter patient survival. To clarify the role of desmosomes in tumorigenesis, further studies regarding their expression and function in primary human cancer tissues are required.

To date, the roles of DSG1 and DSG2 in EHCC are not sufficiently identified. Thus, to analyze the clinicopathological significance and prognostic significance of DSG1 and DSG2, we investigate the difference in their expression in surgically resected specimens among normal bile duct tissues, bile duct adenoma tissues, peritumoral tissues, and EHCC tumour tissues by immunohistochemical staining and study their relationship to prognosis.

## 2. Material and Methods

### 2.1. Sample Collection

The Ethics Committee for Human Research of the Second Xiangya Hospital, affiliated with Central South University, has approved this retrospective cohort investigation. This study is exempt from informed consent since it is a retrospective study, and the data collection and analysis were carried out without disclosing patients' identities. A total of one hundred EHCC tumour tissues, thirty peritumoral tissues, ten biliary tract adenoma, and fifteen normal biliary tract tissues were collected from the 2nd and 3rd affiliate education hospitals of the Central South University between January 2001 and December 2014. All samples obtained from the patients were histologically confirmed by two pathologists. The clinical grade was determined in accordance with the standard TNM classification of malignant tumour 7th edition. Tumours were classified according to the World Health Organization tumour classification system. Histopathologic subtypes of tumour differentiation degrees were determined in accordance with the standard of the World Health Organization as previously described. We collected the survival information of the 100 patients with EHCC via letter or telephone interviews. The follow-up time was 30 months, and patients who survived over 30 months were included in the analysis as censored cases.

### 2.2. Main Reagents

The EnVisionTM Detection Kit was purchased from Dako Laboratories (CA, USA). Rabbit anti-human DSG1 and DSG2 polyclonal antibodies were purchased from Dako Corporation (Carpentaria, CA, USA).

### 2.3. Immunohistochemistry

Immunohistochemical staining of DSG1 and DSG2 was presented by the EnVision TM Detection kit in accordance with the user guide. The EnVisionTM Detection Kit provided positive controls. Briefly, four-micron sections were sliced from routinely paraffin-embedded tissues. After dewaxing, the slices were incubated with 3% H_2_O_2_ in the dark for 15 min, and antigen (heat-induced epitope) retrieval was induced with sodium citrate buffer (10 mm sodium citrate, 0.05% Tween 20, pH 6.0) at 96°C for 30 min. Then, the slices were incubated with rabbit anti-human DSG1 (1 : 100 dilution) and DSG2 primary antibodies (1 : 100 dilution) for 2 hr at room temperature, after rinsing with PBS for 3 × 5 min. The slices were incubated with HRP-conjugated anti-rabbit second antibody for 30 min, followed by DAB substrate staining and haematoxylin counterstaining. At last, slices were dehydrated, soaked in xylene, and mounted with neutral balsam. An average of the percentage of positive cells was calculated from 500 cells in 10 random fields by 2 investigators independently. Slices with ≥25% positively stained cells in the cytoplasm were determined to be positive, while others were determined negative.

### 2.4. Statistical Analysis

Data were analysed using SPSS 17.0 (statistical package for the Social Sciences, Version 17.0). Chi-square test or Fisher's exact test was used for analysing the interrelationship of DSG 1 and DSG2 with histological or clinical factors. The Kaplan-Meier univariate survival analysis and log-rank tests were used for analysing the overall survival of patients with EHCC. Multivariate analysis was performed with the Cox proportional hazards model to calculate 95% confidence interval. A probability level of *P* < 0.05 was considered statistically significant.

## 3. Results

### 3.1. Characteristics of Patients

The clinicopathological data for 100 cases with EHCC are summarized in Table 1. Among them, 39 cases were obtained from female patients and 61 from male patients (M/F = 1.56) with an average age of 58.8 ± 10.2 (ranged from 35 to 80) years. Surgical patterns include radical surgery (54.0%), palliative surgery (36.0%), and only biopsy (10.0%). Following TNM staging, 35 cases were classified as stage I+II (35%), 38 cases were classified as stage III (38%), and 27 cases were classified as stage IV (27%). Following histopathologic subtypes, 31 cases were classified as well-differentiated (31.0%), 34 cases were classified as moderate-differentiated (34.0%), and 35 cases were classified as poor-differentiated (35.0%). Invasion of peripheral tissue and/or organs occurred in 67 cases (67.0%), and regional lymph nodes were metastasized in 38 cases (38.0%). Additionally, gallstones were found in 31 cases (31.0%). Invasion and lymph node metastases were evaluated in accordance with standard criteria. Survival datum of the 100 patients with EHCC was collected via letters and/or telephone calls. The stated follow-up time was limited to thirty months; if someone survived longer than 30 months, they were included in the analysis as a censored case.

Thirty peritumoral tissues samples consisted of 20 samples from male patients (66.6%) and 10 samples from female patients (33.3%) with an average age of 48.5 ± 9.2 (ranged from 35 to 72) years. Following histopathologic subtypes, the pathological examination showed 12 normal tissues, 8 with mild dysplasia, 6 with moderate dysplasia, and 4 with severe dysplasia.

Ten biliary tract adenoma tissue samples consisted of 4 samples from female patients (40.0%) and 6 samples from male patients (60.0%) with an average age of 46.7 ± 10.2 (ranged from 33 to 70) years. Following histopathologic subtypes, the pathological examination showed 6 simple adenoma tissues, 2 with mild dysplasia, and 2 with moderate to severe dysplasia.

Fifteen normal biliary tract tissues were obtained from donors of liver transplantation and were confirmed as normal biliary tract tissues by pathological examination.

### 3.2. DSG 1 and DSG2 Protein Expression in EHCC, Peritumoral Tissues, Biliary Tract Adenoma, and Normal Biliary Tract Tissues

**Figure 1:**
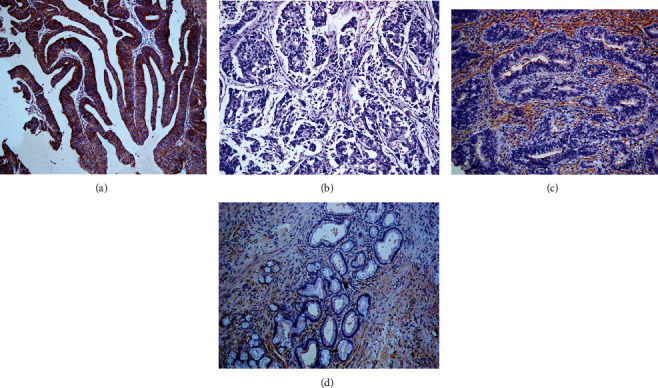
Immunohistochemical staining of DSG1, ×200. (a) Positive expression of DSG1, moderately differentiated EHCC. (b) Negative expression of DSG1, well differentiated EHCC. (c) Positive expression of DSG1, pericancerous tissues. (d) The positive expression of DSG1, adenoma.

**Figure 2:**
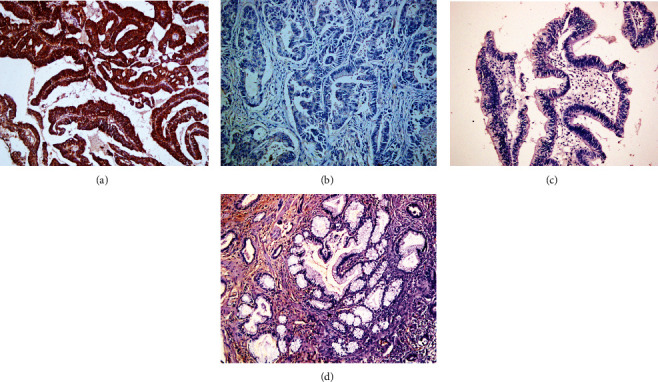
Immunohistochemical staining of DSG2, ×200. (a) Positive expression of DSG2, moderately differentiated EHCC. (b) Negative expression of DSG2, well differentiated EHCC. (c) Positive expression of DSG2, pericancerous tissues. (d) The positive expression of DSG2, adenoma.

Positive DSG1 and DSG2 expressions were observed in the cytoplasm by immunohistochemical staining (Figures 1 and 2). Among the 100 cases with EHCC, positive expression of DSG1 was 42 (42.0%) and of DSG2 was 47 (47.0%), respectively (Table 2). In the thirty peritumoral tissues, positive expression of DSG1 was 22 (73.3%) and of DSG2 was 24 (80.0%), respectively (Table 2). Among the 10 adenoma tissues, positive expression of DSG1 was 8 (80.0%) and of DSG2 was 8 (80.0%), respectively (Table 2). The expression of DSG1 and DSG2 was positive in the whole 15 normal biliary tract tissues. In comparison to peritumoral and normal biliary tract tissues, the positive expression level of DSG1 and DSG2 was downregulated in EHCC (*P* < 0.05) (Table 2). Furthermore, peritumoral tissues and adenoma with positive DSG1 and/or DSG2 expression exhibited moderate to severe dysplasia (Table 2).

### 3.3. Association of DSG1 and DSG2 Expression with Clinicopathological Characteristics of Patients with Extrahepatic Cholangiocarcinoma

We further evaluated the potential correlation between DSG1 and DSG2 expression and clinicopathological parameters of the 100 cases with EHCC. In comparison to poorly differentiated case, advanced TNM stage (III or IV), lymph node metastasis, region invasion, and biopsy only, positive rates of DSG1 and DSG2 expression were both upregulated in cases with the well differentiated type, TNM I + II stage, no lymph node metastasis, radical surgery, and no invasion (*P* < 0.01) (Table 1). The expression of DSG1 and DSG2 presented no significant association with sex, age, tumour sites, and tumour diameter (*P* > 0.05). The relationship between DSG1 expression and DSG2 expression in EHCC was analysed by the *χ*^2^ test. Among the 42 cases with positive DSG1 expression, 30 cases had positive expression of DSG2. Among the 58 cases with negative DSG1 expression, 41 cases had negative expression of DSG2. DSG1 expression was positively correlated with DSG2 expression in EHCC (*χ*^2^ = 17.348, *P* = 0.000) (Table 3).

### 3.4. Correlation of DSG1 and DSG2 Protein Expressions with Overall Survival in Patients with EHCC

**Figure 3:**
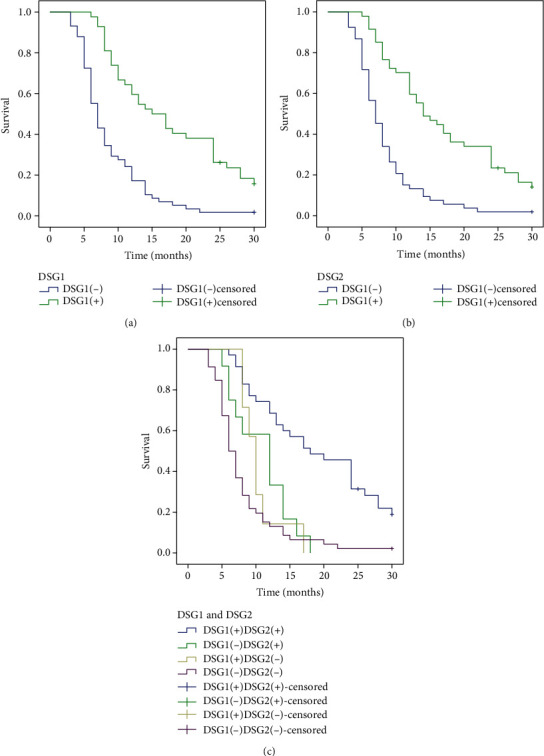
Kaplan-Meier curves for EHCC. (a) Positive and negative expression of DSG1 in EHCC. (b) Positive and negative expression of DSG2 in EHCC. (c) DSG1 and DSG2 expression in EHCC.

Survival data was collected from all EHCC patients. Among the 100 EHCC patients, 58 patients survived less than 12 months, 24 patients survived less than 24 months, 10 patients survived less than 30 months, and 12 patients survived over 30 months; the patients who survived longer than 30 months were analysed as a censored cases. It was discovered that the influential factors, such as differentiation, TNM stage, invasion, lymph node metastasis, and surgical procedure, were closely related to the average overall survival time of the patients with EHCC by Kaplan-Meier survival analysis (*P* < 0.05) (Table 4). The average overall survival time for DSG1 or DSG2 positive patients was significantly higher than that for those with negative DSG1 or DSG2 expression (*P* = 0.000) (Table 4, Figure 3). Furthermore, we defined four groups by the expression of DSG1 and DSG2: positive expression of both DSG1 and DSG2 (+/+), positive DSG1 and negative DSG2 (+/−), negative DSG1 and positive DSG2 (−/+), and both negative (−/−). Kaplan-Meier survival curves revealed that the group with both DSG1 and DSG2 negative expression had the shortest overall survival relative to the other groups, and the group with both DSG1 and DSG2 positive expression had the longest overall survival relative to other groups (Table 4, Figure 3).

### 3.5. Multivariate Analysis

**Figure 4:**
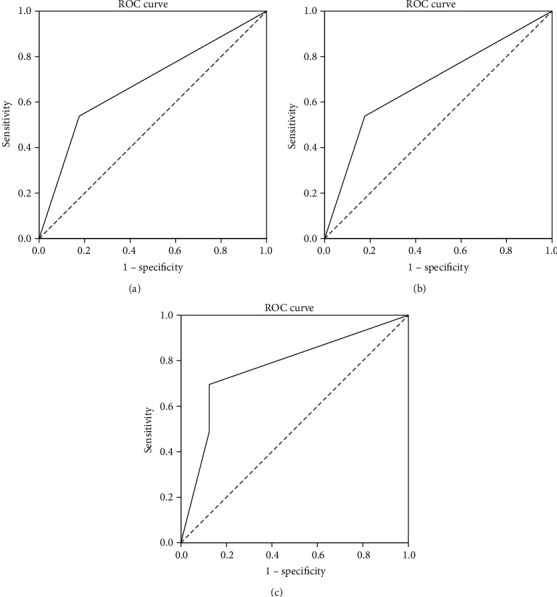
ROC of diagonal segments. (a) ROC of diagonal segments is produced by ties of DSG1 in EHCC. (b) ROC of diagonal segments is produced by ties of DSG2 in EHCC. (c) ROC of diagonal segments is produced by ties of DSG1 and DSG2 in EHCC.

Good differentiation, no lymph node metastasis, no invasion, and a TNM stage of I or II proved to be positively correlated with overall survival and negatively correlated with mortality by Cox multivariate analysis. Positive DSG1 or DSG2 expression positively correlated with overall survival and negatively correlated with mortality. Both DSG1 and DSG2 positive expression proved to be independent prognostic factors (Table 5). The area under the curve of a receiver operating characteristic curve calculated for DSG1 was 0.681 (95% confidence interval: 0.594–0.768), for DSG2 was 0.645 (95% confidence interval: 0.555–0.734), and for DSG1 and DSG2 was 0.772 (95% confidence interval: 0.609-0.936) (Figure 4, Table 5).

## 4. Discussion

EHCC is an aggressive malignancy and has a dismal prognosis. In our research, the average survival time of patients with early TNM stage (I+II) is significantly longer. Furthermore, patients that received radical surgery have longer average survival time. These results demonstrated that early diagnosis is essential to improve the clinical prognosis of EHCC. Additionally, there have been no recent advances in medical therapy for EHCC, so radical surgery remains the best option for meaningful survival. Unfortunately, this dismal prognosis is the result of both the inability to reliably detect EHCC at an early stage and a lack of successful therapies other than radical surgical excision. Most EHCC patients miss their chance to undergo a potentially curable resection because symptoms present late during the course of the disease. Even after an extensive, curative-intent excision of the EHCC, the high rates of liver metastasis and/or local recurrence make treatment efforts nearly futile. Improved knowledge of the molecular and genetic processes that take place in EHCC patients can lead to the development of more efficient prognostic biomarkers as well as new therapeutic targets.

Predictive and prognostic biomarkers are valuable tools in cancer prognosis predicting and therapy monitoring. Although the expression of DSG1 and DSG2 has been proven to be involved in the progression and prognosis of certain types of tumours, the expression and significance of DSG1 and DSG2 in EHCC have not been previously reported. Thus, the frequency of DSG1 and DSG2 proteins was calculated in EHCC tumour tissues, peritumoral tissues, adenoma tissues, and normal biliary tract tissues using immunohistochemical staining to evaluate whether the expression of DSG1 and/or DSG2 had any clinical or pathological significance in EHCC. We observed that the expression of DSG1 and DSG2 in EHCC tumour tissues presented a significant downregulation compared to the nontumour tissues. Negative DSG1 and DSG2 expression is associated with poor differentiation, advanced TNM stages, invasion, metastasis, and poor prognosis of EHCC. To date, this is the first study to report the correlation between DSG1 and DSG2 expression and clinicopathologic characteristics and survival in EHCC patients.

The function of desmosomes is mainly of a mechanical nature, but desmosomes are also integrated in cellular signalling cascades. Desmosome proteins are found to be imbalanced in malignant tumours and seem to play a critical role in invasion and metastasis. However, it is not yet clear if they function as tumour suppressors or as oncogenes. DSG1 and DSG2 are major adhesion structures localized to the cell-cell borders of epithelial cells where they act as cytoplasmic plaque components. Desmosomes also serve as “signalling centres,” playing an active role in modulating several important pathways, including the Wnt/*β*-catenin and the T-cell factor/lymphoid enhancer factor pathways [17]. Mounting evidence supports their participation in modulating cell fate and survival. Desmosomal proteins may activate intracellular signalling through the modulation of expression levels and patterns, both of which can dramatically alter adhesion and cell proliferation [18, 19]. In the interfollicular epidermis, DSG1 and DSG2 are normally expressed at very low level and restricted to the proliferative basal cell layer, leading to tumour cells with high DSG1 or DSG2 expression that often exhibit high malignancy, which is identified by all previous research. Although the potential mechanism needs further study for identification, our findings are consistent with those in previous studies. The positive rates of DSG1 and DSG2 expression were significantly downregulated in EHCC compared with normal tissues. Meanwhile, our study showed that positive rates of DSG1 and DSG2 expression were significantly lower in the case of poorly differentiated types, TNM stages III or IV, positivity of lymph node metastasis, regional invasion, and no resection (biopsy only) (*P* < 0.01), suggesting that DSG1 and DSG2 may be involved in tumorigenesis of EHCC. In addition, negative expression of DSG1 and DSG2 was closely associated with several clinicopathological characteristics of EHCC, which could present aggressiveness and determine the malignant degree of the tumour. The patients with negative DSG1 and DSG2 expression exhibited a shorter survival time than did the patients with positive expression. The dual negative patients had the poorest prognosis. Thus, both DSG1 and DSG2 may inhibit tumour progression in EHCC, and function as prognostic biomarkers. The AUC for DSG1 and DSG2 showed that DSG1 and/or DSG2 may play a role in carcinogenesis, progression, early diagnose, or prognostic prediction of EHCC. Cox multivariate analysis suggested that negative expression of DSG1 and/or DSG2 is an independent prognosis factor for poor prognoses in patients with EHCC. In particular, the predicted value can be significantly improved when DSG1 and DSG2 are combined.

Desmosomal proteins are found to be dysregulated in many types of cancer, and in some, they seem to play a decisive role in invasion and metastasis. EHCC is a highly malignant tumor and poor prognosis with intrinsic resistance to chemotherapy and radiotherapy, resulting that adjuvant therapy is almost ineffective in all patients. Reduced or absent expression of cell adhesion molecules is associated with acquisition of increased invasive and metastatic capacity, consistent with the poorer outcome in the subset of patients with lack of expression of DSG1 and DSG2. Meanwhile, very often, DNA methylation is a common mechanism responsible for gene silencing. DSG2 mRNA expression can be restored by demethylation treatment in some of the lung cancer cell lines [5]. Furthermore, downregulation of DSG2 was significantly associated with promoter methylation, suggesting that DNA methylation is at least partially responsible for the DSG2 gene silencing [5]. Therefore, demethylation treatment maybe an effective therapeutic strategies for EHCC patients with negative DSG1 and/or DSG2, which need further study. We found that negative expression of DSC1 and DSG2 was associated with shorter survival for EHCC patients. The potential up-stream and down-stream mechanism, which have not been fully elucidated, may provide new targets for targeted therapy in the future. Therefore, we can utilize DSG1 and DSG2 as prognostic biomarkers in new therapeutic strategies.

## 5. Conclusions

DSG1 and DSG2 are involved in the carcinogenesis and progression of EHCC. The negative expression of DSG1 and DSG2 was related to poor prognosis in EHCC patients.

## Figures and Tables

**Table 1:** Correlations of DSG1 and DSG2 protein expression with the clinicopathological characteristics of EHCC.

CPC	Case no.	DSG1			DSG2		
		Pos No. (%)	*x* ^2^	*P* value	Pos No. (%)	*x* ^2^	*V*alue
Age (year)							
≤45 years	17	8 (47.1)	0.215	0.643	9 (52.9)	0.290	0.590
>45 years	83	34 (41.0)			38 (45.8)		
Sex							
Male	61	27 (44.3)	0.329	0.566	29 (47.5)	0.018	0.892
Female	39	15 (38.5)			18 (46.2)		
Differentiation							
Well	31	24 (77.4)	23.142	0.000	25 (80.6)	6.434	0.040
Moderately	34	9 (26.5)			11 (32.4)		
Poorly	35	9 (25.7)			11 (31.4)		
Tumour size							
≤3 cm	62	27 (43.5)	0.161	0.689	29 (46.8)	0.003	0.954
>3 cm	38	15 (39.5)			18 (47.4)		
Tumour position							
Hilar site	27	10 (37.0)	0.990	0.609	12 (44.4)	0.991	0.609
Hepatic duct	4	1 (25.0)			1 (25.0)		
Distal duct	69	31 (44.9)			34 (49.3)		
Bile stone							
No	69	33 (47.8)	3.101	0.078	36 (52.2)	2.392	0.122
Yes	31	9 (29.0)			11 (35.5)		
Lymphnode metastasis							
No	62	38 (61.3)	24.924	0.000	39 (62.9)	16.566	0.000
Yes	38	4 (10.5)			8 (21.1)		
Invasion							
No	33	22 (66.7)	12.320	0.000	22 (66.7)	7.648	0.006
Yes	67	20 (29.2)			25 (37.3)		
TNM stage							
I+II	35	23 (65.7)	21.460	0.000	26 (74.3)	27.598	0.000
III	38	17 (44.7)			19 (50.0)		
IV	27	2 (7.4)			2 (7.4)		
Surgery							
Radical	54	32 (59.3)	15.078	0.001	36 (66.7)	19.220	0.000
Palliative	36	9 (25.0)			10 (27.8)		
Biopsy	10	1 (10.0)			1 (80.0)		

DSG1: desmoglein 1; DSG2: desmoglein 2; EHCC: extrahepatic cholangiocarcinoma; Pos No.: positive number.

**Table 2:** Comparison of DSG1 and DSG2 expression in normal tissue, adenoma, peritumoral tissue, and EHCC.

Tissue type	Number of patients (N)	DSG1 positive (%)	DSG2 positive (%)
EHCC	100	42 (42.0)	47 (47.0)
Peritumoral tissues	30	22 (73.7)^∗∗^	24 (80.0)^∗∗^
Adenoma	10	8 (80.0)^∗^	8 (80.0)^∗^
Normal tissues	15	15 (100.0)^∗∗^	15 (100.0)^∗∗^

DSG1: desmoglein 1; DSG2: desmoglein 2; EHCC: extrahepatic cholangiocarcinoma; compared to EHCC: ^∗^*P* < 0.05, ^∗∗^*P* < 0.01.

**Table 3:** The association between DSG1 expression and DSG2 expression in EHCC.

		DSG1		
	DSG2	−	+	Total
	−	41	17	53
	+	17	30	47
Total		58	42	100

*χ* ^2^ = 17.348, *P* = 0.000; −: negative expression; +: positive expression.

**Table 4:** Correlations of clinicopathological characteristics, DSG1 and DSG2 expression with the mean survival in patients with EHCC.

Group	Number of patients (*n*)	Median survival (month)	Log-rank *χ*^2^	*P*
Sex				
Male	61	12.67 (3-30)	0.001	0.980
Female	39	12.59 (4-30)		
Age (year)				
≤45	17	13.82 (3-30)	0.667	0.414
>45	83	12.10 (3-30)		
Differentiation				
Well	31	18.46 (5-30)		
Moderately	34	11.41 (3-30)	27.655	0.000
Poorly	35	7.97 (3-30)		
Tumor size				
≤3 cm	62	12.62 (3-30)	0.235	0.628
>3 cm	38	12.03 (5-30)		
TNM stage				
I+II	35	18.57 (7-30)		
III	38	11.05 (3-30)	57.569	0.000
IV	27	6.26 (3-13)		
Lymph node metastasis				
No	62	15.52 (4-30)	39.001	0.000
Yes	38	7.18 (3-25)		
Invasion				
No	33	17.52 (4-30)	17.399	0.000
Yes	67	9.87 (3-30)		
Surgery				
Radical	54	16.62 (3-30)	48.388	0.000
Palliative	36	7.58 (4-24)		
Biopsy	10	6.90 (3-14)		
DSG1				
−	58	8.66 (3-30)	32.827	0.000
+	42	17.52 (6-30)		
DSG2				
−	53	8.40 (3-30)	31.002	0.000
+	47	16.89 (5-30)		
DSG1 and DSG2				
DSG1(-) DSG2(-)	41	8.087 (3-30)	39.007	0.000
DSG1(-) DSG2(+)	17	10.833 (5-18)		
DSG1(+) DSG2(-)	17	10.429 (8-17)		
DSG1(+) DSG2(+)	30	18.977 (6-30)		

−: negative expression; +: positive expression.

**Table 5:** Multivariate Cox regression analysis of survival rate in patients with EHCC and DSG1 and DSG2 expression.

Groups	Factors	*B*	SE	Wald	*P*	RR	95% CI	
							Lower	Upper
Differentiated degree	Well/moderately/poorly	0.526	0.158	11.083	0.001	1.692	1.241	2.306
Tumour size	≤3 cm/>3 cm	0.515	0.240	4.605	0.032	1.674	1.046	2.679
Lymph node metastasis	No/yes	0.744	0.319	5.440	0.020	2.104	1.126	3.932
Invasion	No/yes	0.785	0.327	5.763	0.016	2.192	1.155	4.162
TNM stage	I/II/III/IV	0.829	0.276	9.022	0.003	2.291	1.334	3.935
Surgery	Radical/palliative/biopsy	0.398	0.191	4.342	0.037	1.498	1.024	2.165
DSG1	−/+	-0.728	0.331	4.837	0.028	0.483	0.252	0.924
DSG2	−/+	-0.672	0.329	4.172	0.041	0.511	0.268	0.973

−: negative expression; +: positive expression; RR: relative risk; CI: confidence interval.

## Data Availability

The data used to support the findings of this study are available from the corresponding author upon request.

## Abbreviations

DSG: Desmoglein
CCA: Cholangiocarcinoma
EHCC: Extrahepatic cholangiocarcinoma
IHCC: Intrahepatic cholangiocarcinoma
TNM: Tumour-node-metastasis
ERCP: Endoscopic retrograde cholangiopancreatography
CPC: Clinicopathological characteristics
Pos No.: Positive number
RR: Relative risk
AUC: Area under the curve
CI: Confidence interval.
